# Comprehensive Management of Inflammatory Bowel Disease: What’s Next

**DOI:** 10.3390/jcm11154584

**Published:** 2022-08-05

**Authors:** Asaf Levartovsky, Uri Kopylov

**Affiliations:** Department of Gastroenterology, Sheba Medical Center Tel Hashomer, Sackler School of Medicine, Tel-Aviv University, Tel Aviv-Yafo 52621, Israel

In the last 20 years, the treatment and management of patients with Crohn’s disease (CD) and ulcerative colitis (UC) have been revolutionized by the introduction of biological therapies and small molecules. Since then, the therapeutic arsenal for these two forms of inflammatory bowel disease (IBD) has increasingly developed, and contemporary treatments include various novel agents targeting different mechanisms. Diagnoses and monitoring of disease activity are progressing as well and are slowly shifting from invasive methods to non-invasive monitoring techniques. In this Special Issue, we focused on providing insights to the broad management of CD and IBD in general, including challenges of therapeutic targets and disease-related complications.

Despite the fact that the advancement of new therapeutic and diagnostic tools has improved the management of IBD, a high percentage of individuals show no clinical benefit post-induction or lose responses over time. Focusing on the group of patients who do not respond to anti-TNF therapy induction (primary non-responders), there is no clear strategy for selection of second-line therapy and the decision is often based on the physicians’ experience. In their review, Gisbert et al. summarized the two main strategies following primary non-response: switching to a second anti-TNF or swapping to an agent targeting a different mechanism of action. In general, a second anti-TNF is less effective when withdrawal to the first anti-TNF is primary failure (36% and 46% remission rate after primary and secondary failure, respectively). However, switching to a different anti-TNF agent may still be beneficial in some patients, even after primary non-response [1]. As for swapping, vedolizumab (VDZ) and ustekinumab (UST) are generally less effective in anti-TNF experienced patients, although a limited number of cases may respond after primary non-response. Nevertheless, major randomized control trials in anti-TNF experienced patients are pertinent to responsibly consider either strategy.

Several real-life studies comparing the effectiveness of VDZ and UST in patients with CD failing anti-TNF agents suggest that the latter could be more effective than the former. However, a loss of response to a subsequent second-class biologic (either VDZ or UST) is common. In addition, studies exploring the effectiveness of both these agents as a third-class biologic after the failure of two previous classes are lacking. A multi-center retrospective cohort study by Albshesh et al. aimed to address this issue in patients with CD [2]. Both inspected groups (VDZ second-class, UST third-class vs. UST second-class, VDZ third-class) achieved similar response rates at weeks 16–22 (circa 56%) and at week 52 (86%), and no differences were observed in clinical remission rates. Both groups had comparable discontinuation rates (17–19%) as well, mostly for a lack of response. These findings demonstrate that third-class biologic therapy can be a practical effective possibility in more than half of the patients with CD, with no differences between VDZ and UST.

Secondary non-responders, patients with lose of response (LOR) over time to anti-TNF agents represent a challenging scenario. In these patients, dose intensification (by increasing the dose or shortening transfusion intervals) is commonly used as a step-up strategy in order to regain therapeutic effect and improve long-term outcomes. Guberna et al. explored the incidence and risk factors for dose intensification following LOR to anti-TNF agents in 173 studies. In their review, dose intensification following LOR occurred mainly during the first year of treatment (2.7–18 months) and was required more frequently in anti-TNF experienced patients, and the findings are correlated with increasing evidence. However, in terms of efficacy, no significant differences in response or remission rates were reported between naïve and non-naïve patients (to infliximab, IFX or adalimumab). Thus, the authors suggest utilizing dose intensification, whether based on therapeutic drug monitoring or not, before switching or swapping [3]. Interestingly, in patients with UC, the dose intensification’s requirement rate was significantly higher compared to CD perhaps due to disease severity and accelerated anti-TNF clearance in active UC, warranting higher drug concentrations.

Anti-TNF drug levels are known to be associated with their therapeutic efficacy. As for VDZ, evidence is emerging of an exposure-efficacy relationship between drug levels and long-term clinical and mucosal outcomes. Whether high drug levels of these agents are associated to potential adverse effects has yet to be established. Veisman and colleagues attempted to explore this association in patients with CD and UC [4]. No specific associations were observed between high-drug concentrations of either IFX or VDZ and specific adverse events, although VDZ levels at some extent (>18 µg/mL) were in fact associated with a higher rate of adverse events. Moreover, severe events were rare and barely justified any additional clinical measures.

The chronic course of IBD and the increasing rates of incidence and prevalence resulted in a burden on healthcare in terms of medical engagement. Due to transmural involvement, patients with CD are at risk of potential intestinal complications including strictures, fistulas and abscesses. A substantial proportion of patients with CD and IBD in general require hospitalization or surgery during their lifetime. A large-scale nation-wide study conducted by Chaparro et al. in Spain showed high cumulative incidences of IBD (16.2%), corresponding with reports in northern Europe [5]. This study demonstrated higher rates of medical therapies and surgical interventions as well as significantly higher hospitalization rates among patients with CD compared to patients with UC.

In addition, discharged patients with CD may re-visit medical attention due to unresolved complaints. This can take a toll on patient well-being and healthcare in terms of delayed diagnosis of complications and economic cost, respectively. Mahajna et al. [6] demonstrated a 60% hospitalization rate of all patients with CD-related complaints presenting to the emergency department (ED) and a considerable 17.4% 30-day re-visit rate of those who were discharged. Notably, the factors associated with returning to the ED were tachycardia and anemia. These clinical features can be just the tip of the iceberg when it comes to complications of IBD.

Acute exacerbations of IBD are events with potential serious consequences and can present unique challenges to the gastroenterologist. Specifically, extraintestinal manifestations such as venous thromboembolism events (VTEs) can complicate admissions. A previous study by our group reviewed a large cohort of patients with IBD and determined the incidence of VTE events and thromboprophylaxis rates in patients hospitalized in a tertiary medical center. Diagnosed VTE rates were similar between patients with an IBD-related and a non IBD-related hospitalization. Albeit non-negligible VTE rates (1.5%), thromboprophylaxis was administered in merely 11.7% of all hospitalizations (IBD and non-IBD related), despite established guidelines, well-known risk factors and implications [7]. In light of these results, perhaps thromboprophylaxis should be considered for non-IBD-related hospitalizations as well. 

Undoubtedly, the chronic course of IBD can impact the mental health of patients, who might suffer from emotional difficulties and adjustment issues that need to be addressed as part of a tailored comprehensive approach alongside medication. The Bonny Method of Guided Imagery and Music is a nonpharmacological music-assisted therapy approach achieving promising emotional improvements in patients with chronic diseases. March-Luján et al. examined the impact of this method on the psychological aspects of IBD patients by using questionnaires and saliva samples of cortisol and IgA levels before and after sessions. Their results demonstrated significant improvements in psychopathologic variables of states of mind (sadness, fear, anger and depression) along with a reduction in saliva cortisol levels, indicating a positive influence on acute physiological stress levels [8].

Overall, the studies in this Special Issue provide a comprehensive view on novel therapeutic strategies in IBD and will assist physicians in improving IBD care and attempt to tailor the right treatment for each patient.
